# *Chlamydia trachomatis:* Management in Pregnancy

**DOI:** 10.1155/S1064744995000378

**Published:** 1995

**Authors:** Alex Allaire, Lawrence Nathan, Mark G. Martens

**Affiliations:** Department of Gynecology and Obstetrics Division of Maternal/Fetal Medicine Emory University School of Medicine 69 Butler street, S.E. Atlanta GA 30303 USA

## Abstract

*Chlamydia trachomatis* is a sexually transmitted disease (STD) commonly diagnosed in pregnancy. *C. trachomatis* has been linked to several pregnancy complications including premature rupture of membranes (PROM), preterm labor and birth, low birth weight, intrauterine growth retardation, and postpartum endometritis. Infants born to mothers through an infected birth canal are at risk for acquiring *C. trachomatis* pneumonitis, conjunctivitis, and nasopharyngeal infection. The standard treatment of *C. trachomatis* in pregnancy is erythromycin. Recently, amoxicillin and clindamycin have been added as alternative regimens for those patients intolerant of erythromycin. This paper reviews the effectiveness and tolerance of the alternative regimens compared with erythromycin and the success of antepartum treatment of chlamydia in preventing the poor pregnancy outcome and neonatal morbidity associated with *C. trachomatis.*


Infectious Diseases in Obstetrics and Gynecology 3:82–88 (1995)
© 1995 Wiley-Liss, Inc.

### *Chlamydia trachomatis*: Management in Pregnancy

**Alex Allaire, Lawrence Nathan, and Mark G. Martens**
*Department of Gynecology and Obstetrics, Emory University School of Medicine, Atlanta, GA*

ABSTRACT

***Chlamydia trachomatis*** **is a sexually transmitted disease (STD) commonly diagnosed in pregnancy. *C. trachomatis* has been linked to several pregnancy complications including premature rupture of membranes (PROM), preterm labor and birth, low birth weight, intrauterine growth retardation, and postpartum endometritis. Infants born to mothers through an infected birth canal are at risk for acquiring *C. trachomatis* pneumonitis, conjunctivitis, and nasopharyngeal infection. The standard treatment of *C. trachomatis* in pregnancy is erythromycin. Recently, amoxicillin and clindamycin have been added as alternative regimens for those patients intolerant of erythromycin. This paper reviews the effectiveness and tolerance of the alternative regimens compared with erythromycin and the success of antepartum treatment of chlamydia in preventing the poor pregnancy outcome and neonatal morbidity associated with *C. trachomatis*.** © 1995 Wiley-Liss, Inc.

KEY WORDS
Antibiotic therapy, neonatal morbidity, sexually transmitted disease, erythromycin, amoxicillin

*Chlamydia trachomatis* is the most common reportable sexually transmitted disease (STD) in the United States.[1,2] *C. trachomatis* is a well-known cause of pelvic inflammatory disease and cervicitis in women. The prevalence of chlamydia in pregnant women ranges from 2 to 30%.[3–5] Certain women are at increased risk: those receiving care at public health clinics, being of young age (<25 years), being of nonwhite race, being of low parity, having multiple sexual partners, having had a new sexual partner in the preceding 3 months, using oral contraception, not using barrier contraception, and having other STDs or a history of other STDs.[3,5,6] In pregnant women, *C. trachomatis* infection carries additional risks compared with nonpregnant women. The risks of *C. trachomatis* in pregnancy and the newborn have recently been reviewed.[3] Although conflicting data exist,[3] maternal *C. trachomatis* infection has several potential antenatal complications including premature rupture of membranes (PROM), preterm labor and birth, low birth weight, intrauterine growth retardation, and stillbirth[3,6–10] as well as postpartum and postabortal endometritis.[11–14] Following passage through an infected birth canal, infants have a significant risk of acquiring *C. trachomatis* pneumonitis (3–18%), conjunctivitis (18–50%), and nasopharyngeal infection (15–20%).[3,15,16] This article reviews the success of the standard treatment regimens in the prevention of these complications and the alternative treatment regimens that have recently been proposed.

### ANTIBIOTIC TREATMENT OF CHLAMYDIA INFECTION

### Current Treatment Recommendations

The present recommendations of the American College of Obstetricians and Gynecologists (ACOG)[5] for screening and treating chlamydia in pregnancy include testing women at their first prenatal visit who are at high risk based on history and physical examination (being <25 years of age, having a

Address correspondence/reprint requests to Dr. Lawrence Nathan, Department of Gynecology and Obstetrics, Division of Maternal/Fetal Medicine, Emory University School of Medicine, 69 Butler Street, S.E., Atlanta, Ga 30303.

Review Article

Received March 17, 1995
Accepted June 30, 1995

history or presence of other STDs, having had a new sexual partner within the preceding 3 months, and having multiple sexual partners), treating again in the third trimester for those thought to be at high risk, and treating those that test positive. The Centers for Disease Control (CDC)[17] recommends similar screening (women <25 years of age and those with new or multiple sexual partners). Routine screening is not recommended. Tetracycline and doxycycline are the recommended agents in nonpregnant women, but these agents are contraindicated in pregnancy because of possible adverse effects on the fetus, including discoloration of deciduous teeth. The most widely accepted antichlamydial regimen in pregnancy is erythromycin base, 500 mg q.i.d. for 7 days, or, if this regimen is not tolerated, 250 mg q.i.d. for 14 days.[1,5]

Erythromycin is commonly thought to have a high rate of intolerance manifested by gastrointestinal (GI) upset, although this side effect has not been universally demonstrated. Alternative regimens have recently been recommended by the ACOG and CDC for those women unable to tolerate erythromycin. The ACOG recommends clindamycin (300 mg orally t.i.d. for 7 days) and amoxicillin (500 mg orally t.i.d. for 7 days), whereas the CDC recommends only amoxicillin as an alternative.

### Alternative Treatment Regimens

### *Clindamycin*

Based on studies reporting success using clindamycin to treat chlamydia in nonpregnant patients, Alger and Lovchik[18] randomly assigned 126 pregnant women between 16 and 24 weeks gestation who had chlamydia cervicitis to receive erythromycin, clindamycin, or placebo. There was no significant difference in the cure rates for those patients treated with clindamycin (92.7%) or erythromycin (83.8%). Erythromycin was associated with significantly more GI complaints than placebo (23.1% vs. 2.4%), whereas clindamycin was not. The patients who experienced side effects were less likely to be compliant and therefore less likely to be cured.

### *Amoxicillin*

Although β-lactam antibiotics were not thought to be effective against chlamydia infections, in vitro[18–21] and nonhuman[22] studies suggested that amoxicillin might be efficacious in the treatment of chlamydia infections in humans. In a small, randomized, double-blind trial comparing amoxicillin, 500 mg b.i.d., with placebo, Bell et al.[23] reported postpartum chlamydial infection in the infant or mother in 4 of 9 of the amoxicillin group and 4 of 6 in the placebo group. This study suggested that amoxicillin was ineffective in the treatment of *C. trachomatis* in pregnant women. Conversely, several studies since have shown that amoxicillin may be an effective treatment. Alexander et al.[24] reported that amoxicillin at a dose of 500 mg t.i.d. has failure rates equivalent to erythromycin. In a prospective, nonrandomized trial, Cromblehome et al.[25] compared the efficacy and patient compliance of treatment with amoxicillin vs. erythromycin. A total of 193 patients with positive cervical cultures were asked to participate in the amoxicillin study. Those who agreed (93) received amoxicillin (500 mg orally t.i.d. for 7 days) and those who refused received erythromycin in the standard regimen. Tests of cure following treatment revealed no significant difference in the percentage cured in the 2 treatment groups ($P > 0.05$). In the amoxicillin group, 98.4% had negative cervical cultures after treatment, compared with 94.8% treated with erythromycin. There was also no significant difference in the rate of vertical transmission as determined by infant culture or antichlamydial IgG titers. Although there was no significant difference in the frequency of side effects, the frequency of stopping the medication because of intolerance of the drug was significantly higher with erythromycin (13% vs. 2%). As this study was neither blinded nor randomized, it may be biased in that the control patients were those who refused entry.

Silverman et al.[26] randomized 74 women who tested positive for chlamydia cervicitis before 36 weeks gestation to receive standard doses of erythromycin or amoxicillin. Test-of-cure cultures were obtained in 3–4 weeks. The cure rate for amoxicillin was 82.3% and for erythromycin was 84.6%. This difference was not significant ($P = 0.91$). Although more patients taking erythromycin experienced side effects (31.6%), all of which were GI disturbances, than those taking amoxicillin (12.8%), this difference was not significant. However, significantly more patients in the erythromycin group than the amoxicillin group stopped the therapy as a result of side effects (18.4% vs 0%).

**TABLE 1. Clinical trials of clindamycin and amoxicillin in the treatment of *C. trachomatis* in pregnancy**

| Author | Study type | Study drug | Control drugs | Treatment results | Drug intolerance |
|---|---|---|---|---|---|
| Alary et al.[28] | Randomized<br>Double-blinded | Amoxicillin | Erythromycin | Amoxicillin—failure 2%<br>Erythromycin—failure 12% | Severe GI side effect—31%<br>Severe GI side effect—6% |
| Alger and Lovchik[18] | Randomized<br>Double-blinded<br>Placebo-controlled | Clindamycin | Erythromycin<br>Placebo | Clindamycin—92% cure rate<br>Erythromycin—83.8% cure rate<br>Placebo—22.7% cure rate | 10%<br>23%<br>2.4% |
| Bell et al.[23] | Randomized<br>Double-blinded | Amoxicillin | Placebo | Amoxicillin—5/9 successfully treated<br>Placebo—2/6 successfully treated | No adverse effects causing discomfort |
| Cromblehome et al.[25] | Nonrandomized<br>Unblinded | Amoxicillin | Erythromycin | Amoxicillin—98.4% cure rate<br>Erythromycin—94.8% cure rate | 2% intolerant<br>13% intolerant |
| Magat et al.[27] | Randomized<br>Double-blinded | Amoxicillin | Erythromycin | Amoxicillin—84.6% cure rate<br>Erythromycin—72.3% cure rate | 7.7% intolerant<br>23.1% intolerant |

Magat et al.[27]conducted a larger double-blind, randomized study comparing amoxicillin and erythromycin for the treatment of *C. trachomatis* in pregnancy. One hundred forty-three women before 36 weeks gestation who had positive cervical cultures were randomized to receive erythromycin base (500 mg orally q.i.d.) or amoxicillin (500 mg orally t.i.d.). Of the women receiving full courses of amoxicillin, 86% were cured as determined by subsequent negative cultures. Of those receiving full courses of erythromycin, 94% were cured. This difference was not significant ($P = 0.14$). Almost half (46.1%) of the patients taking erythromycin complained of nausea and vomiting, and half of these patients (23.1%) discontinued treatment because of the side effects. In the amoxicillin group, only 5 patients (7.7%) experienced side effects, with 1 of these patients (1.5%) discontinuing treatment. If successful treatment is defined as completing the course of medicine and having a negative test of cure, 84.6% of the amoxicillin-treated group and 72.3% of the erythromycin-treated group were successfully treated. The initial power ($\beta = 0.2$) analysis called for 79 patients in each arm; however, upon completion of the study, unexpected results suggested that larger studies would be required to confirm the efficacy of amoxicillin. More recently, Alary et al.[28] found a treatment failure rate of 12% in erythromycin-treated patients vs. 2% in amoxicillin-treated patients in a cohort of 199 patients.

Knowing the relative cost of each medication is useful in prescribing a treatment regimen. The average wholesale cost to the pharmacist of a 7-day course of amoxicillin is $3.36.[29] The equivalent regimen of erythromycin costs $11.76; clindamycin, the most expensive, costs $46.62 for a 7-day course.[29]

Based on the above studies (Table 1) comparing erythromycin with amoxicillin and clindamycin, the ACOG[5] has added amoxicillin and clindamycin as alternative regimens for the treatment of chlamydia in pregnancy for those women who are intolerant of erythromycin therapy. However, the CDC[1] recommends only amoxicillin as an alternate therapy for those patients intolerant of erythromycin. Based on this discrepancy in recommendations, we recommend using amoxicillin for women who are intolerant of erythromycin and clindamycin for patients intolerant of erythromycin and allergic to β-lactam antibiotics.

### Azithromycin

Azithromycin is an azalide antibiotic that, although chemically related to erythromycin, has unique pharmacokinetics. It has excellent in vitro and in vivo activity against *C. trachomatis*.[30,31] The drug is rapidly absorbed by the GI tract and widely distributed to body tissues. It is avidly taken up by cells, resulting in tissue levels that are up to 100 times greater than serum levels. This effect may be especially beneficial in the treatment of chlmaydia, an obligate intracellular pathogen. In addition, migratory leukocytes, which heavily concentrate azithromycin, selectively deliver the drug to sites of inflammation, further increasing the local concentration.[30–33]

Azithromycin, 1 g orally in a single dose, is a recommended regimen for chlamydia cervicitis in nonpregnant women.[1,5] At an average wholesale cost of $24.16/g, azithromycin is approximately twice the cost of erythromycin.[29] The clinical tolerance of azithromycin is superior to erythromycin,[20] and patient compliance may be improved in the pregnant patient. In addition, patient compliance may be further enhanced using single-dose therapy as opposed to multiple-dose regimens for 7–14 days. Reproductive studies with azithromycin in rats have revealed no evidence of impaired fertility or adverse fetal outcome.[34] Although azithromycin is not currently recommended for use in pregnant women, its similarities to erythromycin have led to recent studies on its use in the treatment of endocervical chlamydia in pregnancy. Both Bush and Rosa[35] and Chatterjee and Madar[36] found it to be equal in efficacy to erythromycin in pregnant patients.

Although currently the recommended first-line regimen in the treatment of chlamydia in pregnancy, erythromycin therapy is not ideal because of its high rate of intolerability and its multiple-dose, 1–2 week regimen. Several studies have suggested that amoxicillin or clindamycin therapy may be superior to erythromycin. Larger studies are needed to confirm these results before these agents can be used as first-line agents. Azithromycin may be an efficacious and well-tolerated alternative to erythromycin, but its efficacy and safety in pregnancy are yet to be shown.

### EFFECT OF TREATMENT ON PREGNANCY OUTCOME

The antenatal treatment of chlamydia cervicitis is beneficial in preventing neonatal infection. Schacter et al.[37] treated 184 women with cervical chlamydia infection at 36 weeks gestation with erythromycin. The women who refused treatment served as controls. Significant GI disturbances occurred in 7% of those taking erythromycin, and half of these did not complete the full course of antibiotics. Vertical transmission was seen in 50% of the infants of untreated mothers and in only 7% of the infants of treated mothers. Although this study shows the neonatal benefit from treatment, it is flawed by not being randomized and by using the women who refused treatment as controls. These authors state only that the 2 groups were similar in age and different in racial make-up. It is possible that the 2 groups differed in many more aspects that may be relevant to the transmission of infection from mother to infant. Nonetheless, this study provides some evidence that vertical transmission is preventable.

FitzSimmons et al.[38] followed 33 *C. trachomatis* culture-postive women who received treatment at 36 weeks gestation. Of these 33, 18 received a 7-day course of erythromycin. The remainder of the group failed treatment for a variety of reasons. Of the group who failed treatment, 3 of the newborns were cultured for *C. trachomatis* and 2 of these were culture positive. None of the infants of the women who were adequately treated had positive cultures for *C. trachomatis* or had symptomatic conjunctivitis or pneumonitis. However, this study was not randomized and is plagued by low numbers and poor follow-up. Black-Payne et al.[39] had similar findings using a rapid enzyme immunoassay antigen detection system (Chlamydiazyme) to screen asymptomatic women in the third trimester. No significant differences were noted in the incidence of respiratory-tract illnesses or conjunctivitis in the Chlamydiazyme-negative and Chlamydiazyme-positive treated women. Of those treated with erythromycin, all of whom completed the course of antibiotics, 16% experienced GI side effects. This study was not randomized, and the controls were Chlamydiazyme-negative women who received no antibiotics. The antibiotics could have had a beneficial effect on neonatal outcome that was unrelated to chlamydia infection. In addition, there was no mention of a power analysis, and there exists the possibility of a $\beta$ error.

Although flawed in many respects, the above studies[37–39] suggest that maternal treatment with erythromycin prior to delivery can prevent the vertical transmission of *C. trachomatis*. It is unlikely that a randomized, double-blinded, placebo-controlled study of the efficacy of prenatal treatment of chlamydia cervicitis in the prevention of neonatal infection will be performed. Such a study would be deemed unethical, given the existing evidence of neonatal transmission and the ability to prevent transmission with antimicrobial therapy.

Early studies demonstrated that endocervical chlamydia infections may result in prematurity and increased perinatal mortality.[10–16,37–42] Other studies have not confirmed these findings.[43,44] More recent investigations have suggested that these differences may be a result of recent infection as demonstrated by IgM-positive pregnant patients[44–46] or coinfections such as trichomoniasis.[44]

Just as the results of studies of the relationship between *C. trachomatis* and pregnancy outcome remain controversial, several studies investigating the effect of antenatal treatment of chlamydia cervicitis on the incidence of poor outcomes such as PROM, low birth weight, perinatal mortality, and preterm labor yield conflicting results. In a retrospective review of medical records, Cohen et al.[8] compared 244 pregnant women with chlamydia cervicitis who were successfully treated with 79 patients with chlamydia cervicitis who were unsuccessfully treated and 244 chlamydia-free control patients. The frequency of premature delivery, PROM, premature contractions, and small-for-gestational-age infants was significantly lower in the successfully treated vs. the unsuccessfully treated patients, but was not significantly different from the control patients. No significant difference in the frequency of postpartum endometritis was noted in any of the groups. The major weakness of this study was the use of treatment failures as controls. Although the successfully treated and the unsuccessfully treated were matched for age, marital status, race, socioeconomic status, gravidity, and parity, there remain many other potential confounding variables in the 2 groups that may be risk factors for the outcomes studied.

Ryan et al.[6] prospectively followed 2,433 chlamydia-culture-positive and 9,111 culture-negative pregnant women. Of the *C. trachomatis*-infected women, 1,110 remained untreated throughout the pregnancy, whereas 1,323 received treatment until subsequent cultures were negative. There was a significant increase in the incidence of PROM and low-birth-weight infants and a decrease in neonatal survival in the untreated group compared with the treated group. No such differences were noted in the treated group compared with the culture-negative group. No significant difference in the incidence of chorioamnionitis was found in any of the groups. This study provides evidence that the treatment of *C. trachomatis* prior to delivery decreases the incidence of poor pregnancy outcome. The strength of this study over the previous study by Cohen et al.[8] is that the women who tested positive but were not offered treatment served as controls, as opposed to using those who failed treatment as controls. However, its major weakness is that it was not randomized, and the determinant of treatment was based on the time period of enrollment of women for prenatal care. Although unlikely, as stated by the authors, the differences in outcome between the 2 *C. trachomatis*-positive groups could be related to changes in patient demographics or unrelated institutional treatment policies during the two periods.

In a double-blind, randomized, placebo-controlled trial, Alger and Lovchik[47] found that the eradication of *C. trachomatis* failed to reduce the incidence of PROM, preterm birth, low birth weight, or maternal infectious morbidity compared with controls, although patients with preterm PROM were more likely to have been infected with *C. trachomatis*.

### CONCLUSIONS

These studies provide evidence that the treatment of chlamydia in pregnancy with erythromycin is efficacious in preventing the poor pregnancy outcome linked to chlamydia infection and the transmission of chlamydia to the newborn. The major drawback to erythromycin therapy is its high frequency of GI side effects (23.1–46.1%) and subsequent patient discontinuation of therapy because of the side effects (13–23.1%).[18,25–27] Alternative regimens, including amoxicillin and clindamycin, have been recommended by the ACOG and CDC. These regimens should be reserved as second line for those who have demonstrated intolerance of erythromycin, as no study with adequate power has proved that they are equal or superior in efficacy to erythromycin.

### REFERENCES

1. Centers for Disease Control: 1993 sexually transmitted diseases treatment guideline. MMWR (S-6):S633–S644, 1993.
2. Sweet RL, Gibbs RS: Chlamaydial infections. In: Infectious Diseases of the Female Genital Tract. 2nd ed. Baltimore: Williams & Wilkins, pp 45–74, 1990.
3. McGregor JA, French JI: *Chlamydia trachomatis* infection during pregnancy. Am J Obstet Gynecol 164:1782–1789, 1991.
4. Faro S: *Chlamydia trachomatis* infection in women. J Reprod Med 30:273–278, 1985.
5. American College of Obstetricians and Gynecologists: Gonorrhea and chlamydial infections. ACOG Tech Bull No. 190, March 1994.
6. Ryan GM, Abdella TN, McNeeley SG, Baselski VS, Drummond DE: *Chlamydia trachomatis* infection in pregnancy and effect of treatment on outcome. Am J Obstet Gynecol 162:34–39, 1990.

7. Gravett MG, Nelson HP, Derouen T, Critchlow C, Eschenbach DA, Holmes KK: Independent associations of bacterial vaginosis and *Chlamydia trachomatis* infection with adverse pregnancy outcome. JAMA 256:1899–1903, 1986.
8. Cohen I, Veille JC, Calkins BM: Improved pregnancy outcome following successful treatment of chlamydial infection. JAMA 263:3160–3163, 1990.
9. Martin DH, Koutsky L, Eschenbach DA, Daling JR, Alexander ER, Benedetti JK, Holmes KK: Prematurity and pernatal mortality in pregnancies complicated by maternal *Chlamydia trachomatis* infections. JAMA 247: 1585–1588, 1982.
10. Martius J, Krohn MA, Hillier SL, Stamm WE, Holmes KK, Eschenbach DA: Relationships of vaginal *Lactobacillus* species, cervical *Chlamydia trachomatis*, and bacterial vaginosis to preterm birth. Obstet Gynecol 71:89–95, 1988.
11. Moller BR, Ahrons S, Laurin J, Mardh P: Pelvic infection after elective abortion associated with *Chlamydia trachomatis*. Obstet Gynecol 59:210–213, 1982.
12. Giertz G, Kallings I, Nordenvall M, Fuchs T: A prospective study of *Chlamydia trachomatis* infection following legal abortion. Acta Obstet Gynaecol Scand 66:107–109, 1987.
13. Wager GP, Martin DH, Koutsky L, Eschenbach DA, Daling JR, Chiang WT, Alexander ER, Holmes KK: Puerperal infectious morbidity: Relationship to route of delivery and to antepartum *Chlamydia trachomatis* infection. Am J Obstet Gynecol 138:1028–1033, 1980.
14. Cytryn A, Sen P, Chung HR, Raina S, Cooper R, Louria DB: Severe pelvic infection from *Chlamydia trachomatis* after cesarean section. JAMA 247:1732–1734, 1982.
15. Alexander ER, Harrison HR: Role of *Chlamydia trachomatis* in perinatal infection. Rev Infect Dis 5:713–719, 1983.
16. Schachter J, Grossman M, Sweet RL, Holt J, Jordan C, Bishop E: Prospective study of perinatal transmission of *Chlamydia trachomatis*. JAMA 255:3374–3377, 1986.
17. Centers for Disease Control: Recommendations for the prevention and management of *Chlamydia trachomatis* infections, 1993. MMWR 42:53, 1993.
18. Alger LS, Lovchik JC: Comparative efficacy of clindamycin versus erythromycin in eradication of antenatal *Chlamydia trachomatis*. Am J Obstet Gynecol 165:375–381, 1991.
19. Ridgeway GI, Owen JM, Oriel JD: The antimicrobial susceptibility of *Chlamydia trachomatis* in cell culture. Br J Vener Dis 54:103–106, 1978.
20. Martens MG, Faro S, Phillips LE: Beta-lactam antibiotic and *Chlamydia trachomatis*. Antimicrob News 4(3): 20–23, 1987.
21. Martens MG, Faro S, Maccato M, Riddle G, Hammill H, Wang Y: In-vitro susceptibility testing of clinical isolates of *Chlamydia trachomatis*. Infect Dis Obstet Gynecol 1(1):40–45, 1993.
22. Kramer MJ, Cleeland R, Grunberg E: Activity of oral amoxicillin, ampicillin and oxytetracycline against infection with *Chlamydia trachomatis* in mice. J Infect Dis 139:717–719, 1979.
23. Bell T, Sandstrom I, Eschenbach D, et al.: Treatment of *Chlamydia trachomatis* in pregnancy with amoxicillin. In Mardh PA, Holmes KK, Oriel JD, Piot D, Schachter J (eds): Chlamydial Infections. Fernstrom Foundation Series, Vol 2. Amsterdam: Elsevier, pp 221–224, 1982.
24. Alexander ER, Harrison HR, Lewis M, Sim DA, Podgore JK: Strategies for prevention of infant chlamydial disease. In Mardh PA, Holmes KK, Oriel JD, Piot D, Schachter J (eds): Chlamydial Infections. Femstrom Foundation Series, Vol 2. Amsterdam: Elsevier, pp 225–228, 1982.
25. Cromblehome WR, Schachter J, Grossman M, Landers DV, Sweet RL: Amoxicillin therapy for *Chlamydia trachomatis* in pregnancy. Obstet Gynecol 75:752–756, 1990.
26. Silverman NS, Sullivan M, Hochman M, Womack M, Jungkind DL: A randomized, prospective trial comparing amoxicillin and erythromycin for the treatment of *Chlamydia trachomatis* in pregnancy. Am J Obstet Gynecol 170:829–832, 1994.
27. Magat AH, Alger LS, Nagey DA, Hatch V, Lovchik JC: Double-blind randomized study comparing amoxicillin and erythromycin for the treatment of *Chlamydia trachomatis* in pregnancy. Obstet Gynecol 81:745–749, 1993.
28. Alary M, Joly J, Moutquin JM: Erythromycin, a useful option in *Chlamydia trachomatis*. Lancet 344:1461–1465, 1994.
29. Sanford JP, Gilbert DN, Sande ME (eds): Sanford guide to antimicrobial therapy. Antimicrobial Therapy, Inc., 62, 25th ed, Dallas, 1995.
30. Stamm WE: Azithromycin in the treatment of uncomplicated genital chlamydial infections. Am J Med 91(Suppl 3A):19S–22S, 1991.
31. Johnson RB: The role of azalide antibiotics in the treatment of chlamydia. Am J Obstet Gynecol 164:1794–1796, 1991.
32. Gladue RP, Bright GM, Isaacson RE, Newborg MF: In vitro and in vivo uptake of azithromycin (CP-62, 993) by phagocytic cells: Possible mechanism of delivery and release at sites of infection. Antimicrob Agents Chemother 33:277–282, 1989.
33. Foulds G, Shepard RM, Johnson RB: The pharmacokinetics of azithromycin in serum and tissues. J Antimicrob Chemother 25(Suppl A):73–82, 1990.
34. Hopkins S: Clinical toleration and safety of azithromycin. Am J Med 91(suppl 3A):40S–45S, 1991.
35. Bush MR, Rosa C: Azithromycin and erythromycin in the treatment of cervical chlamydial infection during pregnancy. Obstet Gynecol 84:61–63, 1994.
36. Chatterjee MS, Madar D: Poster: *Chlamydia trachomatis* in pregnancy and efficacy of treatment with erythromycin. Infectious Disease Society for Obstetrics and Gynecology Meeting, Monterey, CA, September 1994.
37. Schachter J, Sweet RL, Grossman M, Landers D, Robbie M, Bishop E: Experience with the routine use of erythromycin for chlamydial infections in pregnancy. N Engl J Med 314:276–279, 1986.

38. FitzSimmons J, Callahan C, Shanahan B, Jungkind D: Chlamydial infections in pregnancy. J Reprod Med 31: 19–22, 1986.
39. Black-Payne C, Ahrabi MM, Brocchini JA, Ridenour CR, Brouillette RM: Treatment of *Chlamydia trachomatis* identified with Chlamydiazyme during pregnancy—Impact on perinatal complications and infants. J Reprod Med 35:362–367, 1990.
40. Martin DH, Koutsky L, Eschenbach DA et al.: Prematurity and perinatal mortality in pregnancies complicated by maternal *Chlamydia trachomatis* infection. JAMA 247: 1585–1588, 1982.
41. Gravett MG, Nelson HP, Derouen T, Critchlow C, Eschenbach DA, Holmes KK: Independent associations of bacterial vaginosis and *Chlamydia trachomatis* infections with adverse pregnancy outcome. JAMA 256:1899–1903, 1986.
42. Ryan GM Jr, Abdella TN, McNeeley SG, Baselski VS, Drummond DE: *Chlamydia trachomatis* infection in pregnancy and effect of treatment on outcome. Am J Obstet Gynecol 162:34–39, 1990.
43. Harrison HR, Alexander ER, Weinstein L, Lewis M, Nash M, Sim DA: Cervical *Chlamydia trachomatis* and *Mycoplasma hominis* infection in pregnancy. Epidemiology and outcomes. JAMA 250:1721–1727, 1983.
44. Sweet RL, Landers DV, Walker C, Schachter J: *Chlamydia trachomatis* infection and pregnancy outcome. Am J Obstet Gynecol 156:824–833, 1987.
45. Berman SM, Harrison HR, Boyce WT, Haffner WJ, Lewis M, Arthur JB: Low birth weight, prematurity, and post-partum endometritis. Association with prenatal cervical *Mycoplasma hominis* and *Chlamydia trachomatis* infection. JAMA 257:1189–1194, 1987.
46. Hardy PH, Nell EE, Spence MR, et al.: Prevalence of six sexually transmitted disease agents among pregnant inner-city adolescents and pregnancy outcome. Lancet 2:333–337, 1984.
47. Alger LS, Lovchik JC: Effect of antenatal treatment of *Chlamydia trachomatis* on pregnancy outcome (Abstract). Presented at the Annual Meeting of the Infectious Disease Society for Obstetrics and Gynecology, Quebec, Canada, August 3–5, 1989.
